# Effect of pericapsular nerve group block and suprainguinal fascia iliaca block on postoperative analgesia and stress response in elderly patients undergoing hip arthroplasty: a prospective randomized controlled double-blind trial

**DOI:** 10.1186/s12871-024-02604-8

**Published:** 2024-07-02

**Authors:** Xiaozhen Cui, Zhi Cheng, Tianyu Zhang, Hai Xu, Hengfei Luan, Jiying Feng, Xiaobao Zhang, Pin Zhu

**Affiliations:** 1 Department of Anesthesiology, Graduate Training Base of Lianyungang First People’s Hospital of Jinzhou Medical University, Lianyungang, Jiangsu People’s Republic of China; 2 Department of Anesthesiology, The First People’s Hospital of Lianyungang, No. 6 East Zhenhua Road, Lianyungang, Jiangsu People’s Republic of China

**Keywords:** Nerve block, Elderly patients, Postoperative analgesia, Hemodynamics, Stress response

## Abstract

**Background:**

As a novel regional analgesic technique, ultrasound-guided pericapsular nerve group (PENG) block has some potential advantages, and we designed a randomized clinical trial (RCT) to investigate whether the ultrasound-guided PENG block combined with general anesthesia can better reduce stress response, maintain intraoperative hemodynamic stability, and reduce postoperative analgesia in elderly hip arthroplasty compared with ultrasound-guided suprainguinal fascia iliaca block (SIFIB) combined with general anesthesia.

**Methods:**

Seventy-four subjects were enrolled over an 8-month period (20 April 2023 to 31 December 2023). All patients were divided into the test group (group P) and the control group (group S) using the envelope as the randomization method. The test group was treated with preoperative ultrasound-guided PENG block analgesia combined with general anesthesia and the control group was treated with preoperative ultrasound-guided SIFIB analgesia combined with general anesthesia. The primary outcome selected was the patient Visual Analogue Scale (VAS) score at 12 h postoperatively.

**Results:**

After generalized estimating equations (GEE) analysis, there was a statistically significant difference in the main effect of postoperative VAS score in group P compared with group S (*P* = 0.009), the time effect of VAS score in each group was significantly different (*P* < 0.001), and there was no statistically significant difference in the group-time interaction effect (*P* = 0.069). There was no statistically significant difference in the main effect of intraoperative mean arterial pressure (MAP) change (*P* = 0.911), there were statistically significant differences in the time effect of MAP in each group (*P* < 0.001), and there were statistically significant differences in the interaction effect (*P* < 0.001).

**Conclusions:**

In summary, we can conclude that in elderly patients undergoing hip fracture surgery, postoperative analgesia is more pronounced, intraoperative hemodynamic parameters are more stable, and intraoperative stress is less induced in patients receiving SIFIB than in patients receiving PENG block.

The incidence of hip fractures increases with age, reaching up to 7% in people aged 75 to 84 years within 10 years, and hip fractures account for 23.79% of total body fractures in the elderly over 65 years of age. Artificial femoral head replacement or total hip replacement is currently the most common treatment for hip fractures. Spinal anesthesia is a common anesthetic technique in hip fracture surgery, and in a study by Neuman et al. [1], it was pointed out that the elderly receiving spinal anesthesia did not recover better than those receiving general anesthesia after surgery, and the incidence of hypotension in patients increased due to the gradual increase in the dose of local anesthetics [2]. On the other hand, hip arthroplasty is often associated with severe pain during the perioperative period. This excessive stress response may easily lead to significant hemodynamic fluctuations in patients, while elderly patients have a variety of underlying diseases. Intraoperative hemodynamic instability will increase the probability of multi-system and multi-organ-related complications [3], not only increasing perioperative risks but also having potential risks for long-term prognosis [4, 5]. In a study by Guerra et al., persistent pain was considered to significantly increase the risk of delirium, cognitive dysfunction, sleep disturbance, and anxiety in elderly patients undergoing hip fracture repair [6]. Therefore, effective perioperative analgesia can reduce pain and surgical trauma-related stress response and maintain intraoperative hemodynamic stability in elderly patients, which can greatly promote postoperative recovery and improve prognosis.

Ultrasound-guided nerve block is an indispensable part of multimodal analgesia program for fracture patients. At present, the commonly used regional analgesia techniques for hip fracture pain management include ultrasound-guided femoral nerve block, fascia iliaca compartment block, lumbar plexus block, etc. Among them, ultrasound-guided suprainguinal fascia iliaca block (SIFIB) is considered to obtain a more satisfactory analgesic effect than other nerve block methods [7, 8]. However, due to the complex nerve distribution in the hip, motor block and incomplete block still occur.

In 2018, GIron-Arango [9] et al. first proposed an ultrasound-guided pericapsular nerve group (PENG) block with local anesthetics injected into the musculofascial plane between the psoas tendon anteriorly and the pubic ramus posteriorly. As a novel regional analgesic technique, PENG block has some potential advantages [10, 11], such as a more precise and complete block range covering the sensory nerves innervating the hip, providing more effective regional analgesia, and no significant quadriceps dyskinesia was observed on this basis, facilitating preoperative turning and leg muscle tension exercises, as well as early postoperative rehabilitation. At present, there are relatively few randomized controlled trials (RCTs) comparing PENG block with SIFIB in reducing the occurrence of stress response and maintaining intraoperative hemodynamic stability and postoperative analgesic effect in elderly patients, so we designed an RCT to investigate whether ultrasound-guided PENG block combined with general anesthesia can better reduce stress response, maintain intraoperative hemodynamic stability, and reduce postoperative analgesia in elderly hip arthroplasty compared with ultrasound-guided SIFIB combined with general anesthesia.

## Design

Our protocol had been approved by the Medical Ethics Committee of the First People's Hospital of Lianyungang. The study protocol conforms to the Declaration of Helsinki. The trial had been registered before enrolment at the China Clinical Trials Registry (ChiCTR2300070518) on 14 April 2023. Seventy-four subjects were enrolled over an 8-month period (20 April 2023 to 31 December 2023). Confirmed informed consent has been obtained from all subjects.

### Patients

Eligible patients must meet all of the following inclusion criteria to be enrolled in the study: 1) the first patient scheduled for elective total hip arthroplasty each day; 2) patients with American Society of Anesthesiologists (ASA) grades of I to III; 3) aged 60 to 75 years old, male or female; 4) patients or their families have been informed of the trial methods and possible adverse reactions and signed an informed consent form; and 5) patients have no relevant contraindications to nerve block. Patients with any of the following could not be enrolled in this study: 1) patients with multiple injuries at other sites; 2) patients with a history of allergy to local anesthetics and neurological diseases; 3) patients with severe skin damage and infectious lesions in the ultrasound scan area; 4) patients with severe heart disease and respiratory diseases; 5) patients with severe liver and kidney dysfunction; 6) patients with abnormal cortisol secretion diseases; 7) patients with mental disorders or emotional and mental retardation and cannot cooperate with.

### Randomization

All patients were divided into the test group (group P) and the control group (group S) using the envelope as the randomization method, the test group was treated with preoperative ultrasound-guided pericapsular nerve group (PENG) block analgesia combined with general anesthesia; the control group was treated with preoperative ultrasound-guided suprainguinal fascia iliaca block (SIFIB) analgesia combined with general anesthesia. Both groups underwent all ultrasound-guided nerve block procedures by the same unblinded investigator who participated only in the randomization and nerve block procedures. Patient screening, informed consent process, and data collection were performed by blinded investigators.

### Process

Heart rate (HR), blood pressure (BP), mean arterial pressure (MAP), first Visual Analogue Scale (VAS) score, as well as the patient's age, height, weight, and sex were recorded the day before surgery. Anesthetic protocols and tests were standardized for all patients. All patients were forbidden to eat for 8 h and drink for 2 h before surgery, and no premedication was given. After admission, venous access was opened and electrocardiogram, noninvasive blood pressure testing, invasive arterial blood pressure testing, continuous MAP monitoring, peripheral oxygen saturation (SpO^2^), and bispectral index (BIS) were monitored.

**Fig. 1:**
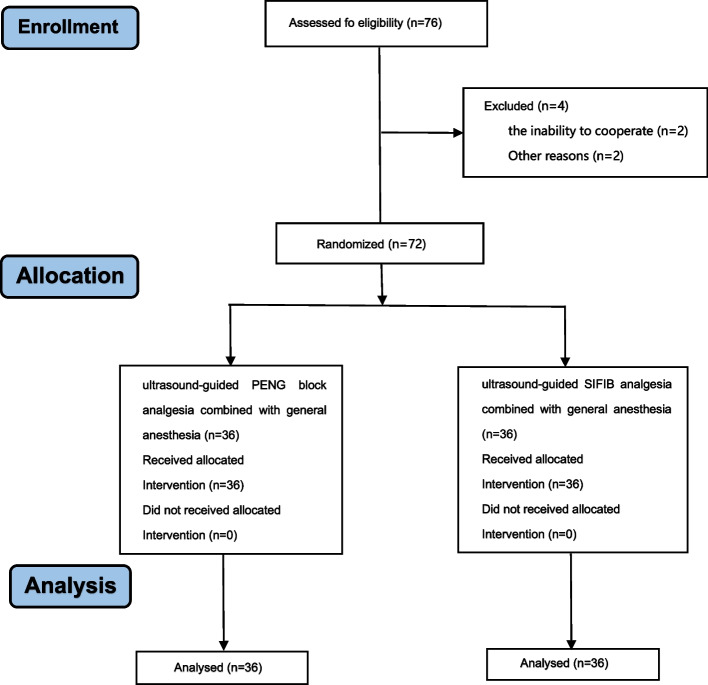
Ultrasound imaging of PENG block. The dashed line is outlined by the arrows. FA indicates femoral artery; PS, psoas tendon; AIIS, anterior inferior iliac spine; IPE, iliopubic eminence

After admission, patients underwent nerve blocks before induction of anesthesia, and all nerve block procedures were performed by the same experienced anesthesiologist who was not involved in the experimental study. Ultrasound-guided PENG block method (Fig. 1): 20 mL of 0.3% ropivacaine was used in this study. A portable ultrasound machine (Sonosite, USA) was applied to scan the target with a probe frequency of 6 to 13 MHz. After the drape was disinfected, the ultrasound probe was fully coated with coupling agent and placed in a sterile probe sleeve. Under aseptic conditions, the low-frequency ultrasound probe was first placed on the transverse plane of the anterior inferior iliac spine. Then it rotated parallel to the pubic branch to obtain short-axis images of the iliopsoas muscle and tendon on the pubic branch located near the iliopubic eminence. After local anesthesia infiltration of the skin, a 23G and 70 mm block needle was inserted into the plane from outside, and the needle tip was placed on the musculofascial plane between the psoas tendon and the ascending pubic branch. After aspirating with no blood and gas seen, 20 mL of 0.3% ropivacaine was slowly injected under intermittent aspiration and continuous ultrasound monitoring to ensure adequate fluid diffusion. When the psoas tendon is pushed slightly upward, it marks adequate fluid diffusion.

**Fig. 2:**
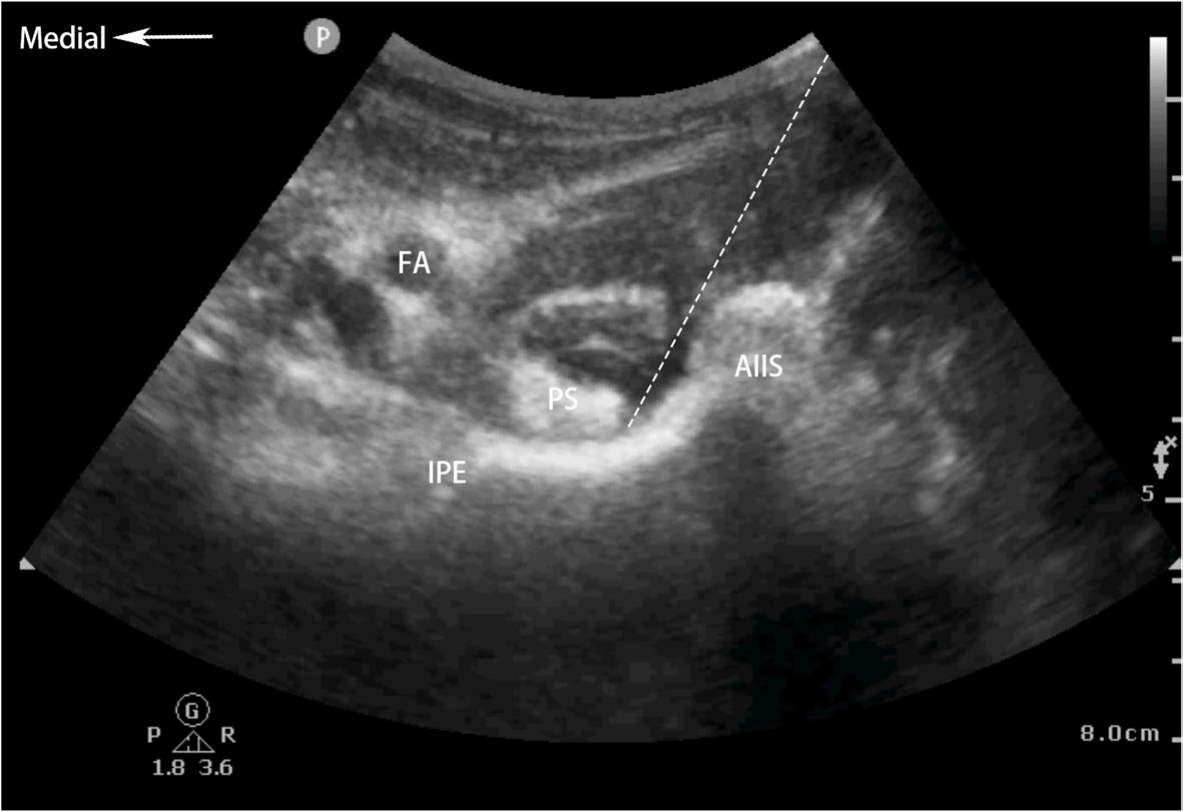
Ultrasound imaging of SIFIB. Dashed line indicates the fascia iliaca; ASIS, anterior superior iliac spine; IM, iliacus muscle; IO, internal oblique; IP, iliopsoas muscle; and TA, transverse abdominus

Ultrasound-guided SIFIB method (Fig. 2): Patients were placed in the supine position with a linear array probe placed at the medial end of the anterior superior iliac spine pointing to the umbilicus to obtain a "hillside" sign to identify sartorius, iliacus, and internal oblique muscles. Using an in-plane technique and caudal-cephalad orientation, the block needle was advanced until its tip was positioned between the internal oblique and iliac muscles below the fascia iliaca. Following negative aspiration, the local anesthetic (40 mL 0.3% ropivacaine) was injected as the needle was slowly advanced toward the cephalad into the fascia iliaca compartment.

### Intraoperative

Anesthesia was induced after preoxygenation with sufentanil 0.4 μg/kg, propofol 1 mg/kg, midazolam 0.05 mg/kg, and cisatracurium besilate 0.15 mg/kg. Mechanically controlled ventilation was conducted after tracheal intubation, VT 6–8 mL/kg, RR 12–16 beats/min, I:E = 1:2, FiO^2^ 100%, oxygen flow 2 L/min, maintained end-tidal carbon dioxide 35–45 mmHg. Anesthesia was maintained with propofol (4.5 mg/kg/h) and remifentanil (0.1–0.3 μg/kg/min). Fluid replacement was performed guided by pulse pressure variation (PPV), and no treatment was given if PPV was ≤ 13% at the measured time points; if PPV was > 13%, 250 mL of compound Ringer's Acetate was rapidly infused and reassessment was performed. If PPV changed significantly (decreased by greater than 2% of the baseline value), compound Ringer's Acetate was continued until the above target was achieved; if PPV did not change significantly (PPV decreased by less than 2% of the baseline value), infusion of the vasoactive drug phenylephrine 0.5 to 5.0 μg/kg · min via intravenous pump was considered until the above goal was met. If the intraoperative blood pressure fluctuated more than 20% of the baseline, or more than 180 mmHg or less than 90 mmHg, the concentration of the depth of anesthesia was adjusted to maintain a BIS value of 40–60, and if the adjustment failed, sufentanil 0.05 μg/kg and urapidil 12.5 to 25 mg was administered for increased blood pressure; norepinephrine 0.03 to 0.1 μg/kg/min was administered for decreased blood pressure. When HR was < 50 beats/min, atropine 0.3 to 0.5 mg was administered; when HR was > 100 beats/min, esmolol 0.5 mg/kg was administered. All drugs above can be repeated if necessary. About 30 min before the end of surgery, sufentanil 0.1 μg/kg and azasetron 10 mg were administered. The cumulative use of propofol and sufentanil was counted after surgery.

Propofol and remifentanil were discontinued at the end of the surgery, the endotracheal tube was removed and the patient was delivered to the PACU after reaching the indications for extubation (the patient was fully conscious and responsive to calls, with the satisfactory recovery of swallowing, choking cough reflex, and respiration (VT > 6 mL/kg)). VAS scores were recorded at PACU admission and PACU discharge, as well as PACU stay. Two milliliters of radial artery blood was drawn 5 min before induction of anesthesia and 5 min after removal of the endotracheal tube, allowed to stand for 1 h, centrifuged at 1000 r/min for 10 min, and the upper plasma was collected and stored in a − 80° C cryogenic refrigerator, and cortisol (Cor) in plasma was measured by ELISA.

MAP and HR were measured and recorded at the following time points: before the nerve block procedure (baseline), 3 min before skin incision, 3 min after skin incision, every 5 min during reaming, 3 min after reaming, and at the last stitch. Analgesic regimen: an electronic patient-controlled intravenous analgesia pump was connected at the end of surgery in all patients, analgesic formula: sufentanil 1.5 μg/L, no background infusion volume, single press volume 2 mL/time, locking time 20 min. Parecoxib sodium 40 mg/dose intravenously was administered when rescue analgesia was required. Bedside follow-up was performed by a blinded investigator on postoperative day 1 (24 h after completion of surgery). Time to first analgesia and cumulative drug use, associated adverse events, and patient satisfaction were recorded.

### Statistical methods

All data in this trial was programmed and calculated using SPSS 20.0 statistical analysis software. Continuous variables were presented as mean ± SD, and median (interquartile range) if normality was not met; t-test or Wilcoxon rank sum test was used for comparison of continuous data between groups. Categorical data was presented as number of cases (constituent ratio), and the chi-square test or Fisher exact test was used to compare categorical data between groups. For repeated measures data, comparisons were made using analysis of covariance or generalized estimating equations (GEE). A *P* value of ≤ 0.05 was considered to indicate a statistically significant difference, with a confidence interval of 95%.

### Sample Size

The primary outcome selected was the patient VAS score at 12 h postoperatively. Sample size calculations were performed using Gpower software. Based on the data of previous studies, patient VAS scores with the PENG block would be 3.01 ± 1.08 and the SIFIB would be 3.91 ± 1.48 after hip surgery. Thirty-four patients per group were required to detect a statistically significant difference with 0.05% alpha and 80% power. Taking into account about 10% of incomplete follow-up or patient dropouts, we recruited a total of 76 patients.

### Outcomes

**Fig. 3:**
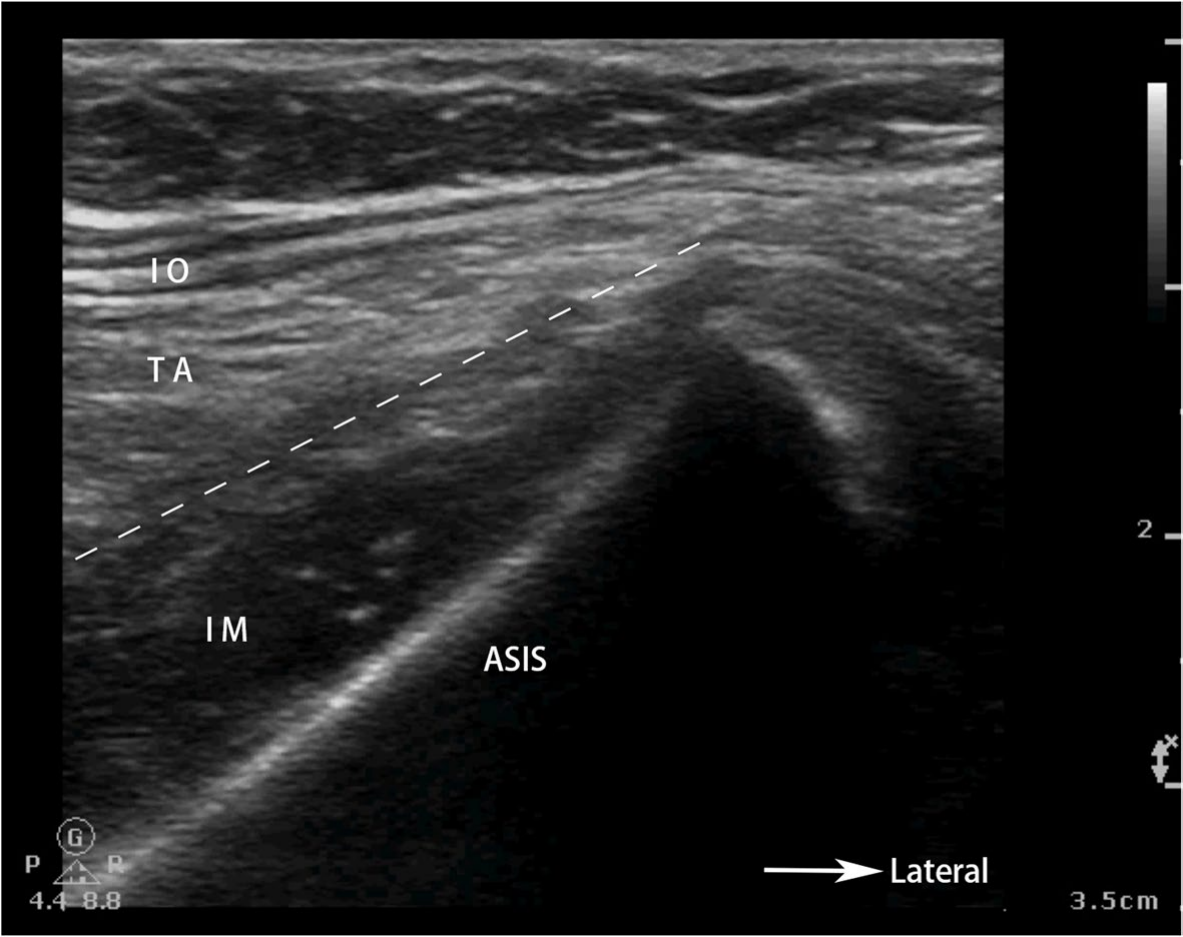
Flowchart of patient selection

A total of 76 patients were enrolled in this study. Two subjects from each group dropped out of the experiment due to the inability to cooperate or operation time exceeding 2 h. Finally, a total of 72 patients who completed the study were included in the statistical analysis, 36 in group P and 36 in group S (Fig. 3). Demographic characteristics of patients in both groups are presented in Table 1, with no apparent statistical difference.

**Table 1:** Demographic characteristics of patients

	Group P	Group S	Effect Value	*P* Value
Age (y)	66.28 ± 4.60	66.47 ± 6.31	0.149	0.882
BMI (kg/m^2^)	23.71 ± 3.26	25.02 ± 3.02	1.773	0.081
Gender, n(%)			2.025	0.155
Male	13(36.1)	19(52.8)		
Female	23(63.9)	11(68.8)		
ASA, n(%)			0.876	0.381
I	2(5.6)	11(30.6)		
II	25(69.4)	13(36.1)		
III	9(25.0)	12(33.3)		
Operation time (min)	71.92 ± 19.05	70.94 ± 12.37	0.257	0.798
Fluid replacement (mL)	1340.28 ± 400.86	1402.78 ± 316.22	0.734	0.465
Preoperative Cor	27.54 ± 26.60	30.84 ± 18.93	0.607	0.546

Values are means ± SD or numbers (percentage)

### Primary outcome: postoperative pain score

**Fig. 4:**
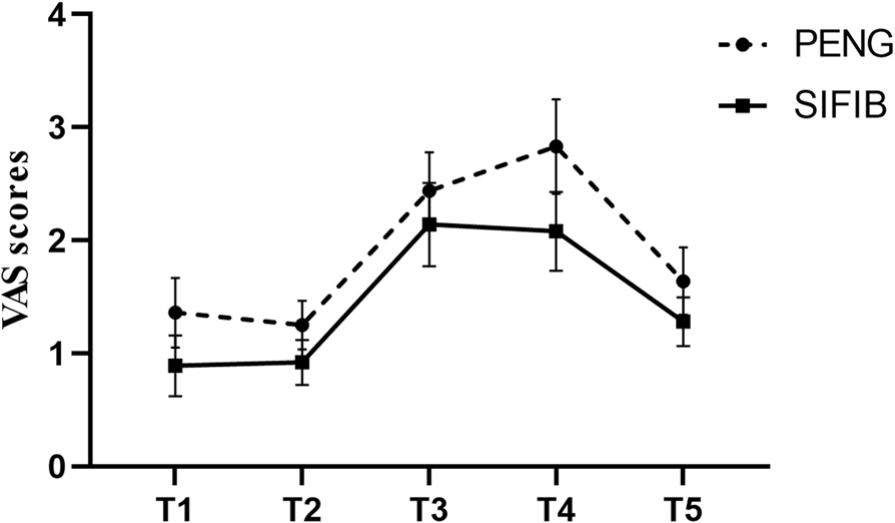
Postoperative Visual Analogue Scale (VAS) scores. T1, PACU discharge; T2, at 1 h postoperatively; T3 at 6 h postoperatively; T4, at 12 h postoperatively; T5at 24 h postoperatively

Postoperative Visual Analogue Scale (VAS) scores are presented in Table 2. There was no statistical difference between the two groups at each time point. After GEE analysis, there was a statistically significant difference in the main effect of postoperative VAS score in group P compared with group S (*P* = 0.009), the time effect of VAS score in each group was significantly different (*P* < 0.001), the group-time interaction effect between the two groups is shown in Fig. 4 and was not statistically different (*P* = 0.069).

**Table 2:** Postoperative Visual Analogue Scale (VAS) scores

Group	VAS scores					Statistics	*P* Value
	PACU discharge	1h	6h	12h	24h		
PENG Group	1.36 ± 1.31	1.25 ± 0.91	2.44 ± 1.44	2.83 ± 1.78	1.64 ± 1.27	25.036	< 0.001
SIFIB Group	0.89 ± 1.14	0.92 ± 0.84	2.14 ± 1.57	2.08 ± 1.48	1.28 ± 0.91	41.20	< 0.001
Statistics	1.629	1.618	0.860	1.943	1.386		
*P* Value	0.108	0.110	0.393	0.056	0.170		

The diference between the two groups at any time point was analyzed based on a generalized estimating equation with a test statistic of Wald chi-square value

### Secondary outcome

#### Hemodynamic parameters

**Fig. 5:**
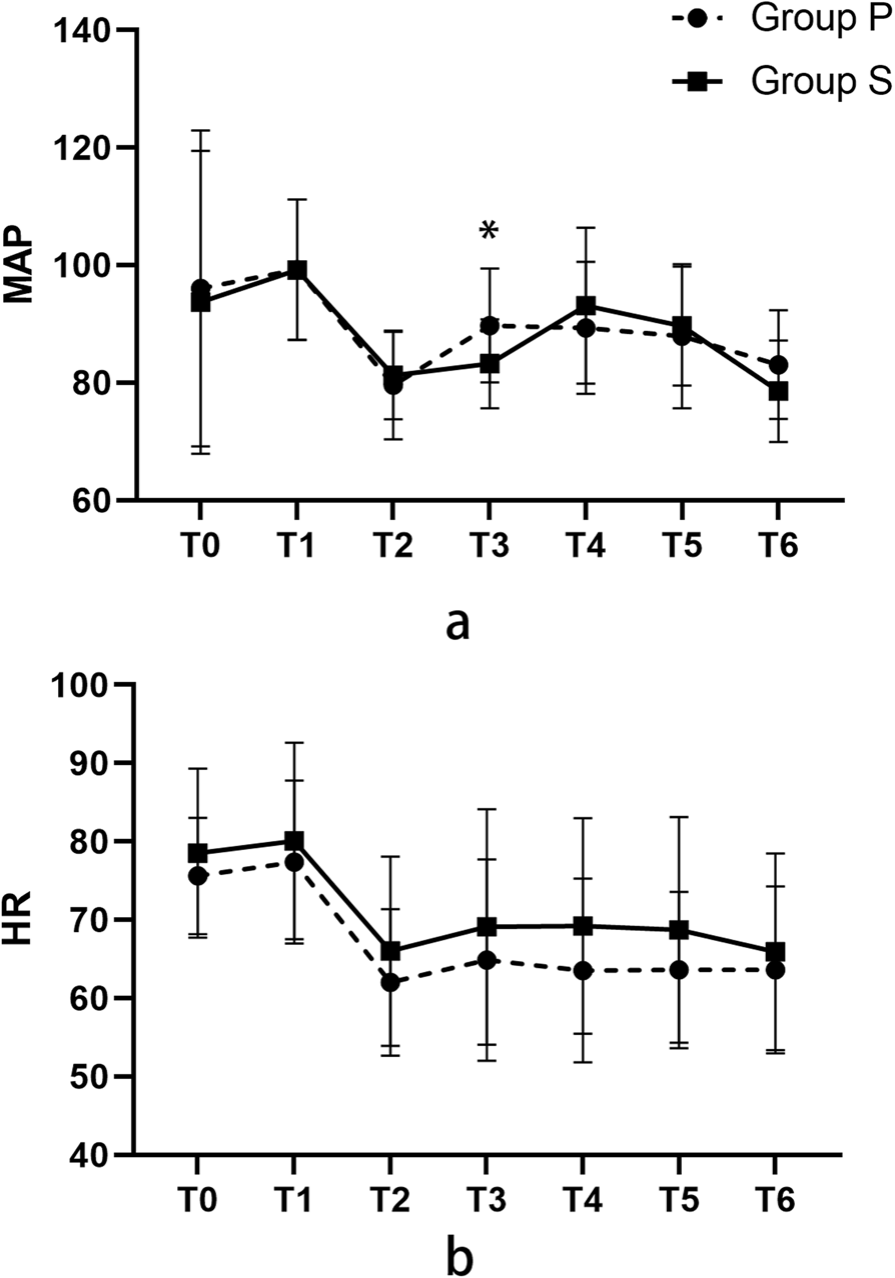
Intraoperative MAPs at each time point in the two groups (**a**), Intraoperative HR at each time point in the two groups (**b**). T0, baseline; T1, 3 min before skin incision; T2, 3 min after skin incision; T3 and T4, every 5 min during reaming; T5, 3 min after reaming; T6, at the last stitch. *P < 0.05 group P compared with group S

Intraoperative hemodynamic changes are presented in Table 3, Table 4 and Fig. 5. MAPs at each time point in the two groups were separately compared and there was a statistical difference at T3. After GEE analysis, there was no statistically significant difference in the main effect of intraoperative MAP change (*P* = 0.911), there were statistically significant differences in the time effect of MAP in each group (*P* < 0.001), and there were statistically significant differences in the interaction effect (*P* < 0.001).

**Table 3:** Intraoperative MAPs at each time point in the two groups

Group	MAP							Statistics	*P* Value
	T0	T1	T2	T3	T4	T5	T6		
PENG Group	96.04 ± 26.85	99.24 ± 11.96	79.60 ± 9.21	89.70 ± 9.70	89.31 ± 11.24	87.92 ± 12.27	83.06 ± 9.24	441.070	< 0.001
SIFIB Group	93.67 ± 25.76	99.19 ± 11.96	81.22 ± 7.42	83.21 ± 7.57	93.12 ± 13.27	89.64 ± 10.13	78.54 ± 8.64	249.165	< 0.001
Statistics	0.378	0.765	2.441	5.670	4.636	2.543	3.707		
*P* Value	0.706	0.727	0.184	0.007	0.117	0.338	0.077		

The diference between the two groups at any time point was analyzed based on a generalized estimating equation with a test statistic of Wald chi-square value. T0, baseline; T1, 3 min before skin incision; T2, 3 min after skin incision; T3 and T4, every 5 min during reaming; T5, 3 min after reaming; T6, at the last stitch

**Table 4:** Intraoperative HR at each time point in the two groups

Group	HR							Statistics	*P* Value
	T0	T1	T2	T3	T4	T5	T6		
PENG Group	75.61 ± 7.42	77.39 ± 10.40	62.03 ± 9.34	64.89 ± 12.84	63.53 ± 11.72	63.61 ± 9.99	63.61 ± 10.66	228.034	< 0.001
SIFIB Group	78.53 ± 10.82	80.08 ± 12.55	66.00 ± 12.08	69.11 ± 15.02	69.22 ± 13.74	68.72 ± 14.39	65.94 ± 12.54	207.676	< 0.001
Statistics	1.334	0.305	1.751	2.503	3.500	3.161	0.016		
*P* Value	0.187	0.884	0.376	0.421	0.178	0.224	0.994		

The diference between the two groups at any time point was analyzed based on a generalized estimating equation with a test statistic of Wald chi-square value. T0, baseline; T1, 3 min before skin incision; T2, 3 min after skin incision; T3 and T4, every 5 min during reaming; T5, 3 min after reaming; T6, at the last stitch

For the comparison of HR between the two groups, after GEE analysis, there was no statistical difference in the main effect of HR change during surgery (*P* = 0.890), there were statistical differences in the time effect of HR in each group (*P* < 0.001), and there was no statistical difference in the interaction effect. (*P* = 0.445).

#### Preoperative and postoperative Cor

After analysis of covariance (Table 5), there was a statistically significant difference in preoperative and postoperative Cor between the two groups after correcting for the baseline effect (correction effect difference 10.83, 95%CI (4.30–17.36), corrected t-value = 3.310, *P* < 0.001).

**Table 5:** Preoperative and postoperative Cor

	Effect Value Mean ± SD	Least square mean difference (95%CI)	t-value	*P* value
PENG Group	4.73 ± 14.21	10.83 (4.30–17.36)	3.310	0.001
SIFIB Group	-7.21 ± 17.20			

*CI* confidence interval

#### Other secondary measures

As shown in Table 6, among other secondary outcomes, there was a statistically significant difference in the cumulative intraoperative remifentanil use between the two groups, while there was no statistically significant difference in the rest.

**Table 6:** Other Secondary Measures

	PENG Group	SIFIB Group	Effect Value	*P* Value
Cumulative intraoperative drug use				
Propofol (mg)	440.81 ± 157.10	431.50 ± 103.99	0.296	0.768
Remifentanil (μg)	726.18 ± 266.73	553.36 ± 132.33	3.950	0.001
Cumulative Opioid Use (μg)	733 ± 6.97	6.08 ± 6.39	0.793	0.430
Number of postoperative rescue analgesia			4.724	0.198
0	8(22.2)	13(36.1)		
1	12(33.3)	15(41.7)		
2	14(38.9)	6(16.7)		
3	2(5.6)	2(5.6)		

## Discussions

Pain management is a critical component of the perioperative management of hip fracture patients following admission. The rational use of nerve block analgesia techniques can bring many benefits to elderly patients [12]. In the perioperative period of hip fracture surgery, anatomical studies have shown that the anterior capsule of the hip joint is the most richly innervated part of the joint, which is mainly from sensory fibers originating from the femoral nerve FN, obturator nerve ON, and accessory obturator nerve AON [13] and is a key target for hip joint analgesia. The posterior capsule is mainly composed of mechanoreceptors and has no sensory fibers [14]. PENG is based on anatomical studies of the hip nerve, setting the nerve related to the anterior capsule of the hip as the main block target, aiming to quickly and accurately relieve hip pain without increasing related adverse reactions.

In this study, the pain scores within 24 h after surgery remained in a low range in both groups, indicating that both block methods were effective in relieving acute pain in the early postoperative period of hip fracture in the elderly. Although there was no significant difference in analgesia between the two groups at separate time points, the overall analysis of multiple measurements showed that SIFIB application resulted in more effective analgesia (main effect *P* < 0.01). In terms of intraoperative analgesia, group S maintained anesthesia with fewer opioids and also showed somewhat better analgesia in patients receiving SIFIB.

From the analysis of intraoperative hemodynamic changes, it was found that there was no significant difference in the interval of intraoperative overall MAP changes between the two groups, but the MAP of patients in the group S at T3 (skin incision) was significantly lower than that in the group P, which explained to some extent that the block effect of SIFIB was more comprehensive compared with the group P, the hemodynamic changes of patients during the operation were more stable, and the stress of patients was reduced.

It should be discussed that combined with the results of statistical analysis of postoperative VAS scores and intraoperative MAP, SIFIB seems to have a more comprehensive and effective analgesic effect than PENG block. This differs from Farag et al.'s [15] meta-analysis. Upon analyzing the reasons for this, on the one hand, SIFIB can effectively block the lateral femoral cutaneous nerve — an effect that PENG block cannot bring. Skin sensation in most hip surgical incisions is innervated by the lateral femoral cutaneous nerve, and perfecting the block of this nerve can effectively reduce the pain produced during skin incision and relieve the pain of the postoperative incision to some extent. On the other hand, it is known from previous anatomical studies that the fascia iliaca compartment (FIC) is a funnel-shaped adipose space between the fascia iliaca and the epimysium of the iliopsoas muscle with superior and inferior openings. Through the openings, the FIC communicates superiorly with the paravertebral space and inferiorly with the adipose space within the femoral triangle. In nerve block techniques, the distribution pattern of local anesthetics is closely related to the volume of injection, and the estimated volume of the FIC in the cadavers was about 23 mL [13]. The SIFIB technique applied in this study is different from the traditional FIC block method, which achieves better hip analgesia by injecting local anesthetics exceeding the volume into this space [16], and the drug can overflow the superior opening of the FIC into the paravertebral space, thereby blocking the ON and other branches of the lumbar plexus [7, 17].

In addition, the changes of plasma cortisol before and after surgery in group S were significantly lower than those in group P. This result also corroborated from the other hand that, to a certain extent, patients receiving SIFIB could obtain better analgesic effect and reduce the degree of intraoperative stress response. There was no difference in the cumulative postoperative opioid consumption and the number of effective analgesic pump presses between the two groups, suggesting that both PENG block and SIFIB can provide satisfactory postoperative analgesia for patients. Besides, because older patients with hip fractures were included in this study, baseline opioid use was lower given the side effects of opioids in the elderly. In this study, no adverse reactions such as puncture site infection and hematoma were observed in the two groups, indicating that both blocks had good safety. However, considering that the PENG block site is close to the hip joint, aseptic principles should be strictly adhered to during surgery to prevent hip joint infection.

### Limitations

There are several limitations to this experiment. First, only the elderly patients aged 60–75 years classified ASAI-III were included in this study. Because of strict patient selection and exclusion criteria, a large number of elderly or frail elderly patients have to be excluded due to complex preoperative underlying diseases or cognitive dysfunction, This to some extent limited the interference of these confounding factors, but may have resulted in a certain selection bias, which means that our results may not be applicable to patients with poorer physical condition. Second, the choice of stress response indicators was relatively single, and the data of postoperative cortisol was only collected at only one time point, which caused a lack of stress evaluations for patients on a basis for a longer time after surgery. Third, because this is a single-center study, the generalizability of the study sample may be compromised. In future studies, we will compare PENG block with sciatic nerve block or other analgesic techniques, and add more stress-related laboratory parameters, so as to investigate the effect of PENG block on patient analgesia and stress more comprehensively.

## Conclusion

In summary, we can conclude that in elderly patients undergoing hip fracture surgery, postoperative analgesia is more pronounced, intraoperative hemodynamic parameters are more stable, and intraoperative stress is less induced in patients receiving SIFIB than in patients receiving PENG block.

## Data Availability

All data generated or analyzed during this study are included in the article. Further inquiries about the datasets can be directed to the corresponding author on reasonable request.

## Abbreviations

PENG: Pericapsular nerve group
SIFIB: Suprainguinal fascia iliaca block
ASA: American Society of Anesthesiologists
HR: Heart rate
MAP: Mean arterial pressure
VAS: Visual Analogue Scale
GEE: Generalized estimating equations
